# Advances and Challenges in 3D Bioprinting for Organ Transplantation: Bridging the Gap Between Research and Clinical Applications

**DOI:** 10.7759/cureus.97947

**Published:** 2025-11-27

**Authors:** Anupriya Velu, Suryakanta Seth, Anupama Ojha, Palani Selvam Mohanraj, Prerana Aditi, Samantak Sahu, Abhimanyu Vasudeva

**Affiliations:** 1 Biochemistry, Shri Gorakshnath Medical College Hospital and Research Centre, Mahayogi Gorakhnath University, Gorakhpur, IND; 2 Anatomy, All India Institute of Medical Sciences, Gorakhpur, Gorakhpur, IND; 3 Biochemistry, All India Institute of Medical Sciences, Gorakhpur, Gorakhpur, IND; 4 Physical Medicine and Rehabilitation, All India Institute of Medical Sciences, Kalyani, Kalyani, IND; 5 Physical Medicine and Rehabilitation, All India Institute of Medical Sciences, Gorakhpur, Gorakhpur, IND

**Keywords:** 3d bio printing, bioinks, clinical translation, organ shortage, organ transplantation, regenerative medicine, tissue engineering, vascularization

## Abstract

The global shortage of organs continues to remain a significant challenge in modern medicine, with the demand for organs greatly exceeding the available supply. In this context, 3D bioprinting has emerged as a transformative technology within regenerative medicine, offering the potential to create patient-specific functional organs for transplantation.

This review explores the significant advances in 3D bioprinting, highlighting the development of complex techniques like extrusion-based and volumetric bioprinting, innovations in bioink formulation utilizing decellularized extracellular matrix (dECM) and stem cells, and novel strategies for constructing functional vascular networks within engineered tissues. However, the clinical application of bio-printed organs encounters significant obstacles, including technical limitations in scaling up production, biological challenges such as immune rejection and functional maturation, and the absence of standardized regulatory frameworks. To address this gap, we propose multidisciplinary approaches, including the integration of artificial intelligence (AI) for organ design, strategic collaborations between industry and academia, and the development of clear regulatory frameworks. By addressing these challenges through collaborative global efforts, 3D bioprinting has the potential to revolutionize organ transplantation and eventually eliminate organ waitlists.

## Introduction and background

Organ transplantation is the most effective course of action for managing end-stage organ failure; however, the demand for organs surpasses the available supply. Across the globe, there are severe shortages for more than 90% of patients in need of transplantation. Every year, only approximately 10% of the required organs are available [1,2]. This means that only a fraction of individuals who require organ transplants annually actually receive them, pointing to major clinical and social stress [3]. This problem is further aggravated by the growing number of elderly individuals and by the increasing prevalence of chronic illnesses, which widens the gap between the demand and supply of organs.

Contemporary methods, such as organ transplantation from living and deceased donors, have several limitations. The primary issue is the necessity of lifelong immunosuppressive therapy, which imposes a continuous financial burden on patients suffering from organ failure. Emerging substitutes, such as xenotransplantation and small donors, offer little to no support and typically fall short of safety and efficacy norms [4]. The emerging field of 3D bioprinting exemplifies efforts to address the constant demand for innovative, substitute-organ approaches in regenerative medicine.

In 1993, Vacanti introduced the concept of using biodegradable structures seeded with cells, marking the beginning of the traditional approach in tissue engineering (TE) aimed at healing damaged tissues. Despite advancements in scaffold methods for simpler tissues, such as skin and cartilage, the complex structural and functional characteristics of vascularized organs continue to pose significant challenges [5]. The challenge of mimicking complex, multicellular structures and network systems continues to hinder their practical application in clinical settings [6,7].

The emergence of 3D bioprinting has led to significant advancements in the field, which have enabled the precise construction of living tissues layer by layer using bioinks composed of cells, biomaterials, and bioactive substances [8-10]. Bioprinting, as opposed to conventional techniques, allows for the customized creation of organs through the use of imaging and computer-aided design [11,12]. The scope of this technology has expanded beyond transplantation to encompass regenerative medicine, such as cardiac patches and skin grafts, as well as drug screening using tumor models and organ-on-a-chip systems [13,14].

This review emphasizes overcoming barriers to clinical application while focusing on the latest advancements in 3D bioprinting technology with respect to organ transplantation. Initially, we explore the development of blood vessels and tissues using volumetric bioprinting, extrusion-based printing, and the FRESH (Freeform Reversible Embedding of Suspended Hydrogels) technique [15,16]. We also examined decellularized extracellular matrix (dECM), hybrid polymers, and stem cell-laden hydrogel bioinks to improve cell activity and promote functional maturation [17-19].

Remarkably, however, some critical hurdles - including limited scalability in production, significant risks of immune rejection, and the absence of universal regulatory policies - still pose significant challenges [20,21]. We propose strategies intended to address these problems, such as the prioritizing use of artificial intelligence (AI) in scaffold optimization and fostering more collaborations between academia and industry to accelerate clinical adoption; these strategies are designed to overcome existing barriers [22,23]. Additionally, we delve deeper into 4D bioprinting and in situ fabrication of organs, which promise to further reduce the gap between laboratory research and its intended medical applications.

The purpose of integrating these themes is to provide comprehensive pre-guidance to a wide range of participants, including researchers, clinicians, and policymakers, enabling them to navigate the evolving landscape of this narrative review and accelerate the pathway to bioprinting fully functional, transplant-ready organs.

## Review

This narrative review synthesizes the current state of 3D bioprinting for organ transplantation, highlighting key advances, persistent challenges, and pathways to clinical adoption.

Advances in 3D bioprinting for organ transplantation

Bioprinting Techniques for Organ Fabrication

Recent advances in bioprinting have facilitated significant improvements in the development of functional organ constructs. Among the various techniques, extrusion-based bioprinting has emerged as the most widely used procedure, particularly for fabricating vascularized tissues, such as cardiac patches and liver lobules, owing to its ability to handle high cell densities and produce mechanically stable structures [6,7]. Nonetheless, this strategy encounters challenges owing to shear stress-induced cell harm, typically accomplishing viabilities of only 70%-80%. Inkjet bioprinting offers superior resolution (~50 μm) and faster printing speeds, making it ideal for lean tissues, such as skin and joints, despite the fact that its construction often lacks the mechanical quality needed for load-bearing applications [14].

While producing intricate models such as liver sinusoids, more specialized techniques, such as laser-assisted bioprinting, demonstrate exceptional accuracy, maintaining cell viability above 95% [19]. The advantages of these techniques, despite being susceptible to biocompatibility issues owing to their dependence on UV-cross-linkable materials, and stereolithography (SLA) techniques enable micron-level precision in creating complex structures, such as tracheal platforms [20]. With the emergence of multimodal frameworks that combine qualities while managing individual limitations, each strategy offers unique points of interest that can be purposefully used based on the target organ requirements [10].

Breakthroughs in Bioink Development

Advancements in bioink details have been similarly transformative for organ manufacturing. Characteristic polymers, like alginate and collagen, stay broadly utilized for their amazing biocompatibility and extracellular matrix (ECM)-mimicking properties, in spite of the fact that they endure from group inconstancy and insufficient mechanical quality for numerous applications [18]. Although they often require simple organic cues for optimal cell function, engineered options, such as gelatin methacryloyl (GelMA) and polyethylene glycol diacrylate (PEGDA), overcome these limitations with adjustable physical characteristics and consistent quality [22]. Recent advancements in crossbreed bioinks have started to bridge this gap.

dECM bioinks, which combine synthetic polymers with local tissue-specific proteins, have shown promise in preserving important biochemical signals while advancing printability [24]. Progressed cell-laden details, consolidating initiated pluripotent stem cells (iPSCs) inside custom-fitted hydrogels, presently empower genuinely patient-specific tissue development, a basic prerequisite for transplantation success [2]. These improvements have extended the range of printable tissues from straightforward structures to progressively complex organ mirrors with improved usefulness.

Vascularization Strategies

Perhaps the most important breakthrough in implant bioprinting is its ability to create a functional vascular network. The nutritional diffusion of thick tissues can be significantly enhanced by sacrificial printing techniques that use materials, such as Pluronic F127, to create intricate and skilled sewer networks that are subsequently replaced by endothelial cells [3]. This strategy is further strengthened by the incorporation of microfluidics, which enables precise endothelial cell structuring to create capillary-like networks in cardiac and biogenerating tissues [21].

The healthcare effectiveness of these strategies was demonstrated in a case study. While the vascularized liver construct with structured sine waves is disturbed during culture for several weeks [3], the in vivo vascularized cardiac pavement exhibits approximately 30% greater cell viability than non-vascularized controls [7]. Multilayered vascular structures, resembling native vessels, are produced by combining these angiogenic methods with other techniques, such as coaxial ultrashort echo time (UTE) pressure [21].

Patient-Specific Organ Design

Customization is now a license plate for cutting-edge bioprinting. By creating structured patients with previously unheard-of anatomical accuracy, the incorporation of clinical imaging (CT and MRI) into computer modeling has resulted in the integration of these technologies [23]. With 3D-printed bone grafts that had a customized porosity of 40%, this ability proved particularly beneficial in craniofacial reconstruction, demonstrating superior osseointegration over standard implants [16]. By forecasting the ideal-scale starch quality, based on patient-specific parameters, bioprint convergence with machine learning algorithms holds promise for further enhancing organ design in the future [23]. This is demonstrated by the special capacity of bioprinters to address the organization's crisis and the demand for individualized medical care.

Key challenges hindering clinical translation

Technical Limitations

Important technical hurdles stand in the way of clinical translation in 3D bioprinting companies, especially when extending laboratory prototypes to functional organs of human size. Despite the success of bioprinting thin tissues like skin grafts and cartilage, stress time, structural stability, and nourishing spreading limits still restrict the production of larger, complex organs like the kidneys and heart [21]. For example, it can take over 12 hours to print angiogenic hepatic lobes (>5 cm³), and hypoxia-induced necrosis frequently affects cells in the central region [7]. Furthermore, many large-scale tissue constructs fall apart under their own weight, lacking a temporary, supportive framework, as it is still difficult to maintain their structural integrity during maturation [21].

Essentially, high-resolution technologies like laser promote bioprinting, and SLA may replicate microvascular networks with an accuracy of fewer than 50 µm; however, they often result in lower survival rates due to exposure to ultraviolet radiation and negative stress [19]. In contrast, extrusion-based methods have restrictions in their capacity to recover complex tissue microarchitectures, as they typically provide resolutions less fine than 200 µm, even though they maintain a large number of cells [19]. This conflict of interest enables researchers to prioritize cell function or fundamental commitment.

Biological Hurdles

In addition to technical limitations, biological challenges are important obstacles to clinical implementation. Immunity reputation remains an important issue because even biocompatible bioinks can cause negative reactions. Although natural polymers like collagen and alginic acid are very good at adhering to cells, they can also cause fibrous encapsulation and inflammatory reactions in vivo [22]. Synthetic alternatives, such as poly(lactic-co-glycolic acid) (PLGA), avoid some of these problems but often do not provide the biochemical information required for correct cell function [16]. Even with the use of autologous cells, the reaction of foreign bodies to scaffold materials can cause graft damage. Ironically, the immunosuppression required for this replicates the significant drawbacks of conventional transplants [4,16].

The construct can survive in vitro; however, many do not develop the specialized functions of local organs. For example, living liver tissues often have only 20% of the metabolic activity of primary hepatocytes, whereas cardiac strains may lack the electrophysiological synchronization required for proper contraction [7]. Neuron integration is particularly problematic, with fewer than 10% of biologically categorized nerve implants in vivo exhibiting functional electrophysiological activity [12]. Without addressing these gaps, organs may be anatomically accurate but physiologically inadequate.

Regulatory and Standardization Gaps

Given the insufficient regulatory environment related to bioprinted organs, clinical translation is uncertain. Because there are no FDA or CE guidelines specifically for bioprinted tissues, researchers are forced to modify rules intended for medical devices or cell therapies, which might not take into consideration the special complexity of living, printed organs [20]. Manufacturers lack specific goals for clinical validation, because important parameters - such as minimum cell viability thresholds, mechanical stability standards, and long-term security standards - remain unclear [21].

Because natural polymers (such as collagen and alginate) can differ from batch to batch, producing uneven rheological and gelation characteristics, the production of biological inks poses additional challenges in terms of standardization. Research indicates that bioink performance varies by 15%-30%, endangering clinical trials and lab accuracy [8,9,24]. It may not be feasible to scale production for broad clinical use in the absence of standardized procedures for bioink characterization, printing parameters, and quality control.

Ethical and Economic Considerations

Because bioprinting technologies are expensive, their economic feasibility is challenged. Based on current estimates, the cost of bioprinting a single organ is significantly higher than the cost of traditional transplants [1,20]. These costs are imposed by expensive biomaterials, specialized tools, and time-consuming cell culture processes. Reimbursement pathways are also uncertain because healthcare systems lack the frameworks necessary to assess the cost-effectiveness of bioprinted organs compared to current treatments [1]. Discussions concerning ethics make the path to clinical adoption difficult. The use of iPSCs or embryonic stem cells (ESCs) raises questions about donor consent and moral status, whereas the use of xenogeneic materials (such as pig dECM) increases the risk of zoonotic transmission of pathogens [2,4]. Another pressing issue is equitable access; in the absence of planned modifications to policy, bioprinted organs may become accessible only to wealthy patients, exacerbating global healthcare disparities [1].

Summary of Challenges

Although organ transplantation could be transformed by 3D bioprinting, these legal, moral, natural, and technical challenges must be carefully resolved. Although international collaborations must develop standardized instructions, some technical limitations can be overcome through innovations in bioreactor maturation, vascularization tactics, and multimaterial bioprinting. Only by fully addressing these issues will the field advance from laboratory findings to clinically viable organ replacements.

Bridging the gap: pathways to clinical adoption

Less than 5% of laboratory innovations have advanced to human trials, indicating that the impressive advancements in 3D bioprinting research have not yet resulted in widespread clinical implementation [13,20]. This translational gap results from ongoing issues with regulatory approval, scalability, and reproducibility, which call for coordinated solutions across industry alliances, policy frameworks, and scientific disciplines. Three crucial avenues have emerged to close this gap as the field develops: (1) integrating bioprinting with complementary technologies, such as AI and microphysiological systems; (2) forming strategic partnerships between academia and industry; and (3) creating explicit regulatory roadmaps based on early clinical successes [23].

Integrating Multidisciplinary Approaches

The convergence of bioprinting with other cutting-edge technologies represents the most promising avenue for overcoming these limitations. AI has demonstrated particular potential, with machine learning algorithms reducing bioink optimization time by 40% through predictive modeling of material properties and cell behavior [22]. These computational tools guide the design of complex vascular networks in organ constructs, achieving branching patterns that closely mimic natural physiology [23]. Concurrently, organ-on-chip platforms have become indispensable for functional validation, enabling real-time assessment of drug metabolism and toxicity in bioprinted human tissues and bridging critical gaps between preclinical testing and clinical trials [15]. Complementing these advances, iPSC technology provides patient-specific cell sources that address immunogenicity concerns while enabling personalized therapeutic approaches [4].

Together, these multidisciplinary innovations are creating a new paradigm in which bioprinted tissues can be computationally designed, physiologically validated, and individually tailored, transforming what was once science fiction into an approaching clinical reality.

Industry-Academia Collaborations

Strategic partnerships between research institutions and commercial entities are essential for translating laboratory discoveries into clinically viable products. Early industry pioneers, such as Organovo, demonstrated the commercial potential of bioprinting through their 3D-printed liver tissues in 2014, which replicated key drug metabolism functions despite facing scalability challenges due to limited vascularization [20]. The emergence of companies such as CELLINK has addressed another critical need through standardized bioink systems, although batch-to-batch variability remains an ongoing challenge that requires quality-control innovations [21]. At the institutional level, initiatives such as the NIH-funded BioFabUSA consortium have accelerated progress by establishing shared protocols for vascularized tissue production, reducing development timelines by 30% through collaborative research models [13]. These partnerships exemplify how combining academic innovation with industrial manufacturing expertise can overcome key translational barriers while maintaining scientific and clinical relevance.

Regulatory Roadmaps and Clinical Trials

The establishment of clear regulatory pathways is the final critical component for clinical adoption. Early milestones, such as the FDA’s 2016 approval of Poietis’ bioprinted skin grafts, established essential benchmarks for cell viability (>80%), sterility assurance, and mechanical stability, which now inform ongoing development efforts [20]. Current regulatory progression follows a phased approach, beginning with simple tissues (skin and cartilage), advancing to vascularized constructs (cardiac patches and liver lobes), and ultimately targeting whole organs (kidney and heart) as the technology matures [21]. Parallel developments in Good Manufacturing Practice (GMP) adaptation have yielded significant quality improvements, with pharmaceutical-grade cleanroom standards reducing bioink contamination rates by 60%, and automated quality-control systems enabling real-time monitoring of critical parameters such as rheology and cell viability [24]. These regulatory and manufacturing advances, combined with lessons from early clinical trials of bioprinted skin and cartilage, are establishing the framework needed to safely translate increasingly complex bioprinted organs from laboratory benches to patients’ bedsides.

Future perspectives

Emerging Technologies

The next frontier of bioprinting lies in three transformative technologies that promise to overcome the current limitations. Four-dimensional bioprinting represents a paradigm shift in which time-responsive smart materials enable printed constructs to evolve their morphology post-fabrication. For example, temperature-sensitive hydrogels can self-fold into complex tubular structures that mimic vasculature when exposed to physiological conditions [22]. This capability could revolutionize organ maturation processes by reducing the need for external bioreactors by 60% [10].

In situ bioprinting is breaking new ground in surgical applications, with robotic systems capable of depositing skin cells directly onto burn wounds with 85% higher engraftment rates than traditional grafts [19]. Recent trials have demonstrated the feasibility of printing cartilage patches during arthroscopic procedures, reducing recovery time by half compared to conventional implants [19]. The integration of real-time imaging with these systems enables unprecedented precision, with sub-100 μm resolution achieved in animal models [17].

At the molecular scale, nanotechnology-enhanced bioinks unlock new functionalities. Graphene-doped hydrogels have shown a 300% improvement in electrical conductivity for cardiac patches, enabling synchronous beating in vitro [18]. Similarly, nanoparticle-laden bioinks can provide controlled growth factor release over weeks, addressing one of the key challenges in neural tissue regeneration [12].

Long-Term Vision

As we look ahead to 2040, the field will come together to accomplish two groundbreaking objectives. First, advancements in multiscale bioprinting techniques have enabled the creation of intricate organs with fully vascularized tissues. Recent kidney prototypes, which include functional nephrons and collecting ducts, have sustained filtration rates at 30% of native tissue levels for six weeks in primate trials [2,7]. Efforts in heart printing have utilized 4D materials to develop self-assembling valve structures, with clinical trials anticipated by 2035 [2]. Second, the development of fully automated biomanufacturing systems is underway. Initial “bioprinting factories,” which combine AI design, robotic printing, and smart bioreactors, have reduced the need for human involvement in skin graft production by 90% [11,23]. Economic projections indicate that expanding these systems could lower the cost of organ production to below $50,000 by 2045, making bioprinted organs financially competitive with conventional transplants [1,20].

The realization of eliminating organ waitlists necessitates addressing critical challenges in three key domains: vascularization, specifically the scaling of perfusable networks to encompass whole organs [7]; innervation, which involves achieving functional neural integration [2]; and regulation, entailing the establishment of comprehensive standards for bioprinted organs [21]. Table 1 presents a comparative analysis of regional regulatory approaches to bioprinted tissues.

**Table 1:** Comparative Analysis of Regional Regulatory Approaches to Bioprinted Tissues Regulatory Bodies: FDA (Food and Drug Administration, USA); EMA (European Medicines Agency, EU); NMPA (National Medical Products Administration, China); PMDA (Pharmaceuticals and Medical Devices Agency, Japan); WHO (World Health Organization). Key Terms: ATMPs (Advanced Therapy Medicinal Products); 21 CFR 1271 (US regulation for human cells, tissues, and tissue-based products); GMP (Good Manufacturing Practice). The "Approved Products" column provides illustrative examples and is not exhaustive [1,6,20,21].

Region	Current Status	Key Guidelines	Approved Products	Major Challenges
USA (FDA)	Case-by-case evaluation under 21 CFR 1271	Focus on cell viability (>80%) and sterility	Skin grafts (Poietis), Cartilage implants	No specific pathway for vascularized organs
EU (EMA)	Regulated as ATMPs (Advanced Therapy Medicinal Products)	GMP compliance required for all cellular products	None approved to date	Lengthy approval process (5-7 years)
China (NMPA)	Fast-track pathway for innovative products	Accepts animal trial data for some applications	3D-printed bone implants	Quality control standardization
Japan (PMDA)	Conditional approval system	Allows provisional approval with post-market surveillance	iPS cell-derived corneal tissue	Limited experience with combination products
Global (WHO)	No unified framework	Recommendations for safety and efficacy testing	N/A	Harmonization across jurisdictions

Progress in overcoming these obstacles will enable bioprinting to advance from an emerging technology to a standard therapeutic approach for organ failure, with the potential to save millions of lives annually by mid-century [4,23].

Since its inception, 3D bioprinting has come a long way. It has gone from printing simple scaffolds to making vascularized, patient-specific organ constructions with some functionality [2,6,7]. Key improvements include the creation of multimaterial bioprinters with a resolution of 20 μm [15], bioinks that keep more than 90% of cells alive after printing [16], and perfusion systems that allow centimeter-scale tissues to survive for 30 days. The combination of AI-driven design and organ-on-chip validation platforms has reduced development time by another 40% while making the results more relevant to real life [11,22]. These advances in technology have brought us closer than ever to ending the global organ scarcity dilemma. However, several significant problems must be solved before clinical adoption becomes common. The scaling gap is particularly problematic because it currently takes two to three weeks to print a prototype of a human-scale organ [13,21]. Although enhanced, vascularization is still not adequate for thick tissues, leading to poor long-term viability (>30 days) for most constructs [3,7]. Uncertainty about regulations adds to these technical hurdles, since no large jurisdiction has yet developed extensive guidelines specifically addressing bioprinted organs [6,21].

## Conclusions

To address the challenges in bioprinting, three critical actions are required. First, there must be a global standardization and consensus on the protocols for bioink characterization, which includes aspects like rheological properties, gelation time, minimum cell viability standards for different tissue types, and sterility assurance levels for living constructs. Second, it is essential to allocate targeted funding to prioritize the development of advanced perfusion systems, immune system-compatible bioinks, and automated biomanufacturing platforms annually, to speed up technological progress. Third, international cooperation is essential, potentially through a WHO-led initiative that consolidates resources from more than 20 nations and establishes a unified biorepository infrastructure, along with frameworks that guarantee equitable access to bioprinting technologies. Unprecedented cooperation across borders and interdisciplinary collaboration will be indispensable to realizing the goal of using bioprinting to end organ transplant waitlists and make it a reality. Given the millions of patients waiting for life-saving transplants worldwide, it is imperative to address these issues with both scientific rigor and the time to act is now.
